# Effect of Olfactory Rehabilitation on the Recovery of Post-Coronavirus Disease Olfactory Dysfunction: A Randomized Controlled Trial

**DOI:** 10.7759/cureus.61855

**Published:** 2024-06-06

**Authors:** Alex Zxi Jian Ho, Nur Izzati B Ishak, Eugene Hung Chih Wong

**Affiliations:** 1 Department of Otorhinolaryngology, International Islamic University Malaysia, Kuantan, MYS; 2 Department of Otorhinolaryngology, Head & Neck Surgery, Faculty of Medicine and Health Sciences, Universiti Malaysia Sabah, Kota Kinabalu, MYS

**Keywords:** olfactory training, post covid-19 complication, smell identification test, mometasone nasal spray, persistent olfactory dysfunction, olfactory rehabilitation, covid-19

## Abstract

Introduction

Persistent olfactory dysfunction was seen in many patients upon coronavirus disease 2019 (COVID-19) infection recovery. However, research on its management was very limited, especially among the Southeast Asian population.

Objectives

We aim to investigate the role of olfactory rehabilitation and topical corticosteroids among post-COVID-19 olfactory dysfunction patients in Malaysia, and at the same time to determine factors leading to olfactory recovery post-COVID-19 infection.

Methods

Adult Malaysians with persistent olfactory dysfunction one month post-COVID-19 recovery were recruited. Thirty-one patients were randomly assigned into three groups with 10 patients being given olfactory training (Group 1), another 10 being given mometasone furoate nasal spray/olfactory training (Group 2), and 11 patients being assigned to the control group (Group 3). All groups were followed up for an average duration of six months. Olfactory function was evaluated by Top International Biotech Smell Identification Test (TIBSIT) scores and Olfactory Disorder Questionnaire (eODQ) prior to randomization, at three and six months after recruitment.

Results

The baseline characteristics of patients were similar in all groups. Generally, patients of all three groups showed a statistically significant improvement in the TIBSIT scores after six months. The TIBSIT scores for Group 2 were statistically significantly higher than the control at three months but not at six months. As for Group 1, no statistically significant differences in TIBSIT scores at both three and six months were noted when compared to control. Statistically significant improvements were seen in the eODQ scores in all three groups.

Conclusion

No superiority of intervention for post-COVID-19 olfactory dysfunction was seen compared to control.

## Introduction

Coronavirus disease 2019 (COVID-19) was first identified in Malaysia back in January 2020 [1]. Common presentations of COVID-19 include fever, cough, dyspnea, myalgia, sore throat, smell, and taste disturbances [2]. In particular, smell disturbances were shown to be strongly associated with COVID-19 infection [3,4]. COVID-19-related olfactory dysfunction was initially believed to be transient. However, the issue of persistent olfactory dysfunction has been commonly described [2,5-8].

In clinical practice, commonly seen treatment modalities for post-viral olfactory dysfunction (PVOD) include oral corticosteroids, nasal steroid spray as well as olfactory rehabilitation/training [9]. The neuronal plasticity properties of the olfactory neurons were postulated to enable one to relearn and identify olfactory stimuli while repetitive stimulation increases neurotrophic factor activity [10,11]. This understanding gave rise to the development of an olfactory training regime for post-infection, post-trauma, and certain neurodegenerative disorders [12]. In relation to post-COVID-19 olfactory dysfunction, Lechien et al. reported significant olfactory improvement for patients who are complaint to olfactory training for at least six months [13]. Another study by Pires et al. showed similar findings after classical olfactory training over a duration of four weeks [14].

As for corticosteroid use, better improvement was illustrated when concurrent steroid administration and olfactory training were used in cases of PVOD [15]. However, conflicting evidence on the role of intranasal corticosteroid was shown. Generally, improvement was seen in patients receiving topical steroids and olfactory rehabilitation; however, no significant differences were noted upon comparison with those receiving olfactory rehabilitation kits only [16,17]. Only one study reported significant improvement in terms of the severity of olfactory loss with the addition of topical corticosteroids [18].

Generally, factors such as the female sex, younger age group (<50 years), and presence of comorbidities such as dyslipidemia were deemed to be significantly associated with olfactory dysfunction after COVID-19 infection [19,20]. Other COVID-19 symptoms such as the presence of fever, sore throat, and loss of appetite were significantly associated with persistent post-COVID-19 olfactory dysfunction [20].

In our study, we aim to investigate and compare the effectiveness of olfactory training against combined treatment (topical corticosteroids/olfactory training) in improving residual smell disturbances among patients after recovery from COVID-19 infection. At the same time, we also aim to investigate the factors associated with post-COVID-19 olfactory dysfunction and its recovery among the Malaysian population. To the best of our knowledge, no similar studies pertaining to this topic were published within the Southeast Asia region.

## Materials and methods

Study design and patients

This study is a prospective randomized controlled study conducted in the city of Kota Kinabalu, Sabah, Malaysia from January to November 2022. The first part of the study involved a retrospective collection of data on COVID-19 patients with olfactory dysfunction.

Residents of Sabah over the age of 18 years with previously confirmed COVID-19 infection (evidenced by positive RT-PCR) and residual olfactory dysfunction for more than four weeks after recovery were recruited into the study. Recovery from COVID-19 infection was taken as the last day of quarantine. Patients with history of prior olfactory symptoms, intranasal pathologies (e.g. nasal polyposis, granulomatous sinonasal pathologies, nasal trauma, sinonasal neoplasm, rhinosinusitis), history of neurodegenerative diseases (central and peripheral nervous system), psychiatric pathology, history of radiotherapy to the head and neck region, prolonged corticosteroid therapy, and inhalational recreational drugs use were excluded from the study. The study protocol received approval from the National Medical Research Register (NMRR) of Malaysia on the 10th of December 2021 with the identification number NMRR-21-901-59536 (IIR). 

All participants signed written informed consent and all proceedings of the study were conducted in accordance with the Helsinki Declaration of 1975. A sample size of 30 randomized patients was calculated to be able to provide 80% power for a significance level of 0.05.

Initial assessment

A comprehensive history was obtained from all patients upon the first consultation. Details collected included age, gender, ethnicity, comorbidities, family history, occupation, alcohol, and smoking history. Other than that, information related to COVID-19 infection such as the onset of symptoms, date of recovery, duration of residual olfactory symptoms, and history of COVID-19 vaccination was obtained. All patients went through thorough clinical assessment and nasal endoscopic examination. Following that, the olfactory assessment was carried out via the Top International Biotech Smell Identification Test (TIBSIT) as well as the English Olfactory Disorder Questionnaire (eODQ) (Appendix).

In total, 947 post-COVID-19 patients were identified from January to April 2022, where a total of 95 patients had residual smell disturbances. Out of those, 53 patients attended the initial consultation for study recruitment, where 13 patients did not fulfill the inclusion criteria and nine patients declined to participate further. The remaining 31 patients were recruited for the study.

Treatment outlines

Patients with residual olfactory dysfunction were then randomized into three groups via a computer-generated sequence:

 i. Group 1: Olfactory training

 ii. Group 2: Mometasone furoate nasal spray and olfactory training

 iii. Group 3: Control group

The olfactory training kits were prepared by allocating 1 mL of essential oils of four different odorants (rose, lemon, cloves, eucalyptus) into 50 mL tightly sealed brown glass jars accordingly. Additionally, cotton pads were inserted into each brown jar to prevent the spillage of essential oils. Patients were advised to sniff each odor for 10 seconds, with a 10-second interval of rest between each odor. This process was repeated for all odors and performed twice a day. Those who received mometasone furoate nasal spray were instructed to administer two puffs (100 μg) once daily. The method and duration of intervention were proven to show improvement of olfactory function in cases of PVOD [21].

Follow-up

Olfactory tests with the TIBSIT and eODQ questionnaire were performed before the randomization, at three months and six months after recruitment to evaluate changes in patients’ olfactory function. The research team was kept blinded throughout the study. Upon each follow-up, olfactory training kits were ensured to have an intact aroma, and new sets were provided if needed. Similarly, patients receiving mometasone furoate nasal spray were given adequate stock throughout the study. No adverse reaction or complication was reported by the intervention given in this study.

Statistical analysis

The Statistical Package for the Social Sciences version 28 (IBM SPSS Statistics 28; IBM Corp, Armonk, NY) was used for statistical analysis. Baseline demographic data were expressed as mean and standard deviation for continuous data or frequency and percentages for categorical data. One-way analysis of variance (ANOVA) tests were used to compare demographic data between the patient groups. The comparisons of various scores before and after interventions were analyzed with the Wilcoxon signed-rank test. A comparison of scores between the control and the intervention groups was done using the Mann-Whitney U test. Fisher’s exact test was used for univariate analysis to look for an association between various factors and outcomes while Spearman’s rank order correlation was used to analyze the correlation between the TIBSIT scores and the eODQ scores. Any results with a p-value less than 0.05 were considered statistically significant.

## Results

Patient demographics and disease characteristics

The patients were randomized into three separate groups according to the intervention received. Group 1 consisted of 10 patients, Group 2 consisted of 10 patients, and Group 3 consisted of 11 patients. No subjects were dropped from the study due to loss of follow-up. Overall, the median age of patients (at the time of the study) was 32 years (mean: 35.9 ± 9.7, range: 20-60) where 67.7% of the patients were females and 32.3% were males. There was no statistically significant difference in age between the three patient groups (p = 0.578). Overall, the majority of the patients were of Kadazan (22.6%) ethnicity followed by Dusun (19.4%) and “Others” (which includes Kedayan, Rungus, and Brunei ethnicity) (58.0%). Most of the patients were non-smokers (87.1%) and did not drink alcohol (87.1%). Besides that, most patients were fit and healthy with ASA Grade I (58.1%), followed by Grade II (35.5%) and Grade III (6.5%).

All patients have smell disturbances as per inclusion criteria. Other most common symptoms experienced by patients were cough (64.5%), followed by fever (58.1%) and shortness of breath (19.4%). Overall, the median duration of symptoms of COVID-19 was 10 days (mean: 12.2 ± 7.2, range: 7-34). The median duration of symptoms of smell disturbances was 60 days (mean: 77.1 ± 61.2, range: 5-180). Most patients required isolation at a quarantine center (Likas Quarantine Centre) (41.9%), followed by home isolation (32.3%) and hospital admission with oxygen support (25.8%). The majority of the patients had two doses of vaccines (64.5%) at the onset of infection, followed by patients who had two doses plus a booster vaccine (32.3%). Only one patient was unvaccinated during the onset of COVID-19 symptoms (3.2%). In terms of the types of vaccine received prior to the onset of infection, most patients had received Pfizer (83.9%), followed by Sinovac (12.9%) vaccines (Table 1).

**Table 1:** Summary of Patient, Disease, and Vaccine Characteristics According to Groups ASA Classification: American Society of Anesthesiologists Classification; COVID-19: coronavirus disease.

Parameters		Group 1	Group 2	Group 3	
		Number of patients (percentage)			
Ethnicity	Malay	0	1 (3.2%)	2 (6.5%)	
	Chinese	0	2 (6.5%)	1 (3.2%)	
	Indian	0	0	1 (3.2%)	
	Kadazan	2 (6.5%)	3 (9.7%)	2 (6.5%)	
	Dusun	4 (12.9%)	0	2 (6.5%)	
	Bajau	1 (3.2%)	2 (6.5%)	2 (6.5%)	
	Others	3 (9.7%)	2 (6.5%)	1 (3.2%)	
Smoking		3 (9.7%)	0	1 (3.2%)	
Alcohol		1 (3.2%)	2 (6.5%)	1 (3.2%)	
ASA Classification	I	4 (12.9%)	7 (22.6%)	7 (22.6%)	
	II	4 (12.9%)	3 (9.7%)	4 (12.9%)	
	III	2 (6.5%)	0	0	
COVID-19 symptoms	Cough	3 (9.7%)	3 (9.7%)		0
	Fever	4 (12.9%)	7 (22.6%)		9 (29.0%)
	Shortness of breath	6 (19.4%)	5 (16.1%)		7 (22.6%)
	Headache	1 (3.2%)	1 (3.2%)		2 (6.5%)
	Sore throat	1 (3.2%)	0		0
Severity of disease	Home quarantine	1 (3.2%)	3 (9.7%)		6 (19.4%)
	Quarantine center without oxygen support	5 (16.1%)	5 (16.1%)		3 (9.7%)
	Hospital admission with oxygen support	4 (12.9%)	2 (6.5%)		2 (6.5%)
		Mean ± standard deviation			
Duration of COVID-19 symptoms (days)		14.8 ± 8.9	12.4 ± 8.0		9.5 ± 3.8
Duration of smell disturbances (days)		75.7 ± 58.9	105.7 ± 60.8		52.4 ± 57.6
Vaccination status	2 doses	6 (19.4%)	8 (25.8%)	6 (19.4%)	
	2 doses + booster	3 (9.7%)	2 (6.5%)	5 (16.1%)	
	No vaccine	1 (3.2%)	0	0	
Vaccine types	Pfizer	6 (19.4%)	10 (32.3%)	10 (32.3%)	
	Sinovac	3 (9.7%)	0	1 (3.2%)	

TIBSIT and eODQ scores

Group 1

There was an increase in TIBSIT scores from the first assessment and at both subsequent visits, where the median scores increased from 35 to 37.5 and 38.5 at three and six months, respectively. Only two (20%) patients at three months and four (40%) patients at six months moved category from either anosmia to hyposmia, or from hyposmia to normosmia when compared to the “normative data” for their age and gender. Wilcoxon signed-rank test showed no statistically significant changes at three months (p = 0.06) but there were statistically significant changes in the scores at six months (p = 0.01) follow-ups. There was a decrease in eODQ scores from 63 (first visit) to 53.5 (three months) and 51.5 (six months). There were no statistically significant reductions at three months (p = 0.121) but there were statistically significant changes in the questionnaire scores at six months (p = 0.011). 

Group 2

There was an increase in TIBSIT scores from the first assessment and at both subsequent visits, where the median scores increased from 32.5 to 40.5 and 44 at three and six months, respectively. Four (40%) patients at three months and eight (80%) patients at six months moved the category from hyposmia to normosmia when compared to the “normative data” for their age and gender. Wilcoxon signed-rank test showed statistically significant changes in the values at both three-month (p = 0.005) and six-month (p = 0.001) follow-ups. There was a decrease in eODQ scores from 81.5 (first visit) to 62.5 (three months) and 48 (six months). There were also statistically significant reductions in the questionnaire scores at three months (p = 0.006) and six months (p = 0.001). 

Group 3

There is an increase in TIBSIT scores from the first assessment and at both subsequent visits, where the median scores increased from 38 to 41 and 44 at three and six months, respectively. Three (27.3%) patients at three months and six (54.5%) patients at six months moved the category from hyposmia to normosmia when compared to the “normative data” for their age and gender. Wilcoxon signed-rank test showed statistically significant changes in the values at both three-month (p = 0.002) and six-month (p = 0.002) follow-ups. There was a decrease in eODQ scores from 65 (first visit) to 62 (three months) and 60 (six months). There were also statistically significant reductions in the questionnaire scores at three months (p = 0.013) and six months (p = 0.045). 

Tables 2, 3 describe a summary of TIBSIT and eODQ results for all three groups.

**Table 2:** Summary of TIBSIT Scores TIBSIT: Top International Biotech Smell Identification Test.

	Group 1		Group 2		Group 3	
Visit	Median score (IQR)	p-Value	Median score (IQR)	p-Value	Median score (IQR)	p-Value
First	35 (29-38.75)	-	32.5 (30-35.75)	-	38 (32-38)	-
3 months	37.5 (33.5-45)	0.06	40.5 (39-43.75)	0.005	41 (38-43.5)	0.002
6 months	38.5 (36.25-42.75)	0.01	44 (43-44.75)	0.001	44 (39-44)	0.002

**Table 3:** Summary of eODQ Scores eODQ: English Olfactory Disorder Questionnaire.

	Group 1		Group 2		Group 3	
Visit	Median score (IQR)	p-Value	Median score (IQR)	p-Value	Median score (IQR)	p-Value
First	63 (48.25-79.75)	-	81.5 (70.25-98.5)	-	65 (58-86.5)	-
3 months	53.5 (45-68.5)	0.121	62.5 (37.5-81.75)	0.006	62 (44.5-83)	0.013
6 months	51.5 (41.25-58.5)	0.011	48 (38.5-80.75)	0.001	60 (42-78.5)	0.045

Comparisons of TIBSIT Scores Between Control and Intervention Groups

For comparisons between Group 1 and Group 3, there were no statistically significant differences in TIBSIT scores at three months (p = 0.973) and six months (p = 0.387). At three months, the improvement in TIBSIT scores for Group 2 was statistically significantly higher than Group 3 (p = 0.036). However, there were no statistically significant differences between the scores at six months (p = 0.085).

Factors Associated With Improvement in TIBSIT Scores

Univariate analysis of various factors revealed no statistically significant associations between patients’ intervention group, age, gender, ethnicity, ASA grade, smoking and alcohol status, COVID-19 symptoms and severity, duration of COVID-19 and smell disturbances, vaccination status and types, with changes (improvement) in their TIBSIT scores at three-month and at six-month follow-ups (all p-values >0.05) (Table 4).

**Table 4:** Univariate Analysis of Various Factors With Improvement in TIBSIT Scores at 3 Months and 6 Months After Intervention ASA Classification: American Society of Anesthesiologists Classification; COVID-19: coronavirus disease.

Factors	TIBSIT scores improvement at 3 months; n (%)	p-Value	TIBSIT scores improvement at 6 months; n (%)	p-Value
Intervention				
Olfactory training	2 (6.5)	0.608	4 (12.9)	0.185
Olfactory training + mometasone furoate nasal spray	4 (12.9)		8 (25.8)	
Control	3 (9.7)		6 (19.4)	
Age				
20-30 years	4 (12.9)	0.539	7 (22.6)	0.450
30-40 years	4 (12.9)		8 (25.8)	
40-50 years	1 (3.2)		1 (3.2)	
50-60 years	0		2 (6.5)	
Gender				
Male	2 (6.5)	0.445	6 (19.4)	0.880
Female	7 (22.6)		12 (38.7)	
Ethnicity				
Malay	1 (3.2)	0.090	3 (9.7)	0.101
Chinese	3 (9.7)		3 (9.7)	
Kadazan	0		2 (6.5)	
Dusun	2 (6.5)		3 (9.7)	
Bajau	1 (3.2)		2 (6.5)	
Others	2 (6.5)		5 (16.1)	
ASA classification				
I	5 (16.1)	0.572	9 (29.0)	0.471
II	4 (12.9)		8 (25.8)	
III	0		1 (3.2)	
Smoking				
Yes	0	0.170	1 (3.2)	0.151
No	9 (29.0)		17 (54.8)	
Alcohol				
Yes	1 (3.2)	0.849	2 (6.5)	0.726
No	8 (25.8)		16 (51.6)	
COVID-19 symptoms				
Shortness of breath	2 (6.5)	0.796	3 (9.7)	0.656
Cough	6 (19.4)	0.873	10 (32.3)	0.220
Fever	4 (12.9)	0.326	9 (29.0)	0.284
Headache	1 (3.2)	0.849	1 (3.2)	0.151
Duration of COVID-19 symptoms				
<7 days	1 (3.2)	0.151	2 (6.5)	0.073
7-14 days	7 (22.6)		14 (45.2)	
14-21 days	1 (3.2)		1 (3.2)	
21-28 days	0		0	
28-35 days	0		1 (3.2)	
Duration of smell disturbances				
<1 month	3 (9.7)	0.970	8 (25.8)	0.980
1-2 months	1 (3.2)		2 (6.5)	
2-3 months	1 (3.2)		2 (6.5)	
3-4 months	2 (6.5)		3 (9.7)	
4-5 months	1 (3.2)		1 (3.2)	
5-6 months	1 (3.2)		2 (6.5)	
COVID-19 severity				
Home quarantine	4 (12.9)	0.361	7 (22.6)	0.640
Isolation center	2 (6.5)		7 (22.6)	
Hospital isolation ward	3 (9.7)		4 (12.9)	
Vaccination status				
2 doses	7 (22.6)	0.562	12 (38.7)	0.489
2 doses + booster	2 (6.5)		6 (19.4)	
Vaccination type				
Pfizer	7 (22.6)	0.517	16 (51.6)	0.445
Sinovac	2 (6.5)		2 (6.5)	

Correlation Between TIBSIT and eODQ Scoring

At three months, there was a strong correlation between the TIBSIT scores and eODQ scores, which was statistically significant (Spearman’s correlation coefficient, rs = -0.454, p = 0.010). Similarly, there was a strong correlation between the TIBSIT scores and eODQ scores, which was statistically significant at six months follow-up (rs = -0.454, p = 0.010).

## Discussion

Considerable interest was given to post-COVID-19 olfactory dysfunction in recent years, especially to investigate potential treatment modalities. To date, studies on the role of olfactory rehabilitation and intranasal steroid therapy in post-COVID-19 olfactory dysfunction remain very limited. Our randomized controlled trial monitored the olfactory status of 31 patients who were all suffering from post-COVID-19 olfactory dysfunction. The demographics of our study participants included ethnicities of minority groups, thus a good reflection of the local demographic population especially in the state of Sabah, Malaysia.

Olfactory rehabilitation was deemed to be an effective treatment modality for PVOD. Its potential role as a treatment for post-COVID-19 olfactory dysfunction has received much interest. In a recent study by Lechien et al., post-COVID-19 patients who adhered to olfactory rehabilitation for at least six months showed significant improvement in olfactory function when compared to control [13]. Yaylaci et al. reported a similar outcome as their study showed significant improvement after olfactory rehabilitation in comparison to control [22].

At the same time, Pires et al. reported improvement in olfactory function after classical olfactory training (by using four different odors) over four weeks [14]. Interestingly, increasing the intensity of olfactory training (by using eight odors) did not yield a significant difference when compared to the classical olfactory training regime [14]. A notable similarity between these studies is the lack of data on COVID-19 vaccination. The only data available was reported by Pires et al. whereby only 68.6% of their study sample received vaccination. This may suggest the possible role of COVID-19 vaccination in facilitating olfactory function return as the majority of our participants were vaccinated prior to the onset of COVID-19 illness (96.8%).

As for intranasal steroid therapy, there were differing opinions on its role in post-COVID-19 olfactory dysfunction. A randomized control study conducted in Egypt showed significant olfactory function improvement in both groups receiving mometasone furoate nasal spray/olfactory rehabilitation and control (olfactory rehabilitation only). However, there were no significant differences upon comparing the two groups. It is worth noting that the mode of olfaction testing used was the visual analog scale (VAS), a non-psychophysical olfactory test [16]. Psychophysical olfactory tests (e.g.: UPSIT, Sniffin’ Stick test) are more accurate in the measurement of olfactory function [23]. Similarly, another prospective longitudinal case-control study performed in Germany described no significant differences in olfactory function recovery between mometasone furoate nasal spray and olfactory training against olfactory training only [17]. Upon investigating the role of mometasone furoate nasal spray as a treatment for post-COVID-19 olfactory dysfunction, Hosseinpoor et al. reported that no significant differences were observed when patients were given mometasone furoate nasal spray or placebo [24].

On the other hand, a study by Kasiri et al. reported that their patients who received a combination of mometasone furoate nasal spray and olfactory training showed better improvement in terms of olfactory loss severity against control (olfactory training only) [18]. However, there were no significant differences in the comparison of the University of Pennsylvania Smell Identification Test (UPSIT) results between groups. Based on these findings, the pathogenesis of COVID-19 olfactory dysfunction may be attributed to neurological causes rather than a sequela of local inflammation. Other forms of corticosteroid treatment such as oral corticosteroids were investigated for their potential as a treatment modality. Several studies on oral corticosteroids showed significant improvement when used in addition to olfactory rehabilitation among patients with post-COVID-19 olfactory dysfunction [25,26].

Olfactory dysfunction often impacts a person’s quality of life considerably. Lechien et al. showed that their participants reported a significant impact of olfactory dysfunction on their activities of daily life (75.4%) and social activities (78.9%) [13]. In our study, the English Olfactory Disorder Questionnaire (eODQ) was used to measure the impact olfactory dysfunction has on the quality of life. It is a validated English version of a German language olfactory disorders questionnaire designed to evaluate the daily life impact of olfactory dysfunction [27]. Generally, all groups reported statistically significant improvement in eODQ scores, which corresponded with the improvement of TIBSIT scores at six months.

In our study, there were no significant correlations between any characteristics with the outcome of post-COVID-19 olfactory dysfunction. A prospective multicenter study in its attempt to identify associations of post-COVID-19 olfactory dysfunction outcomes found no significant related clinical factors [28]. However, other studies have shown statistically significant correlations to post-COVID-19 olfactory dysfunction duration, which included older age, diabetic status, and duration of COVID-19 illness [16]. In Malaysia, factors such as the female sex, the younger age group (<50 years), and the presence of comorbidities such as dyslipidemia were shown to be significantly associated with persistent olfactory dysfunction [19,20].

This study, however, is subjected to several limitations. First, the sample size calculation did not consider the number in each group and drop-out rates as it shows the overall total number of samples for the study. However, our sample size was comparable to other studies in the literature and there were no drop-outs in our cohort. Other than that, covariate matching was not performed upon study initiation. Nonetheless, our result analysis showed that all groups were well-matched in terms of age and ethnicity. In the future, we recommend clinical studies with a larger sample size and longer duration with objective measurement tool to further validate our results. Additionally, future research should take into account the role of vaccination on the outcome of olfactory function recovery after COVID-19 infection. 

## Conclusions

Our study showed a statistically significant improvement in terms of olfactory function across all three groups. However, no superiority was shown in the comparison of the intervention groups for post-COVID-19 olfactory dysfunction with control. This suggests a possibility that patients with post-COVID-19 olfactory dysfunction show improvement in terms of olfactory function and quality of life even without intervention.

## Figures and Tables

**Figure 1:**
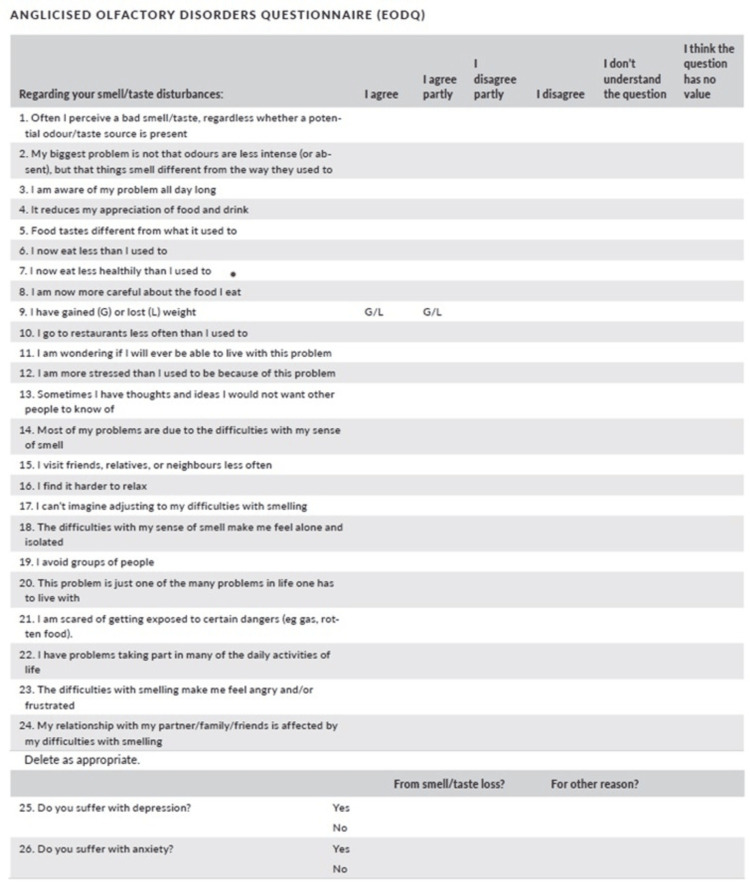
English Olfactory Disorder Questionnaire (eODQ) (Page 1)

**Figure 2:**
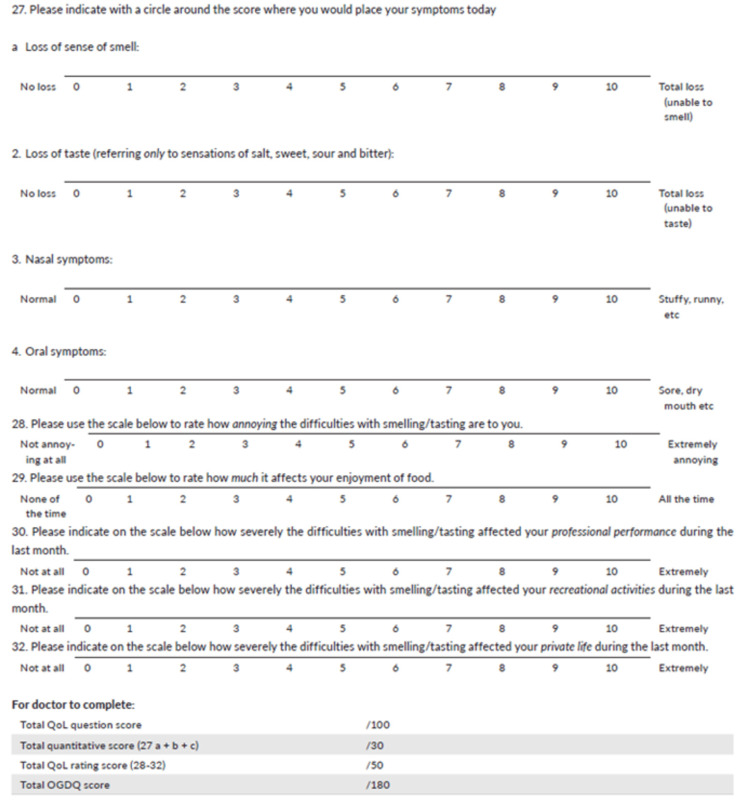
English Olfactory Disorder Questionnaire (eODQ) (Page 2)
